# Primary intraosseous squamous cell carcinoma arising from an odontogenic keratocyst: a case report and literature review

**DOI:** 10.3332/ecancer.2013.316

**Published:** 2013-05-09

**Authors:** Sandhya Tamgadge, Avinash Tamgadge, Neha Modak, Sudhir Bhalerao

**Affiliations:** Department of Oral & Maxillofacial Pathology and Microbiology, Padmashree Dr DY Patil Dental College & Hospital, Sector 7, Nerul, Navi Mumbai, Maharashtra, Pin-400706, India

**Keywords:** primary intraosseous squamous cell carcinoma, odontogenic keratocyst, mandible, Carnoy’s solution, p53

## Abstract

Primary intraosseous squamous cell carcinoma (PIOSCC) derived from an odontogenic keratocyst (OKC) is a rare malignant neoplasm of the jaws, which is locally aggressive with quite poor prognosis. The incidence of carcinomas arising in odontogenic cysts was reported to be approximately 1–2/1000. The number of well-documented cases of PIOSCC *ex* OKC is extremely small; hence, no sufficient incidence data are available in the literature. Overall, the survival rate of an individual, which is a period of two years, is very poor, and this can be attributed to the delayed diagnosis. But knowledge of the histopathological and immunohistological features of PIOSCC allows accurate and early diagnosis of the lesion so that an early and appropriate treatment can be instituted for better prognosis. The following report describes an extremely rare case of PIOSCC of the mandible derived from an OKC in a 20-year-old female patient.

## Introduction

Carcinoma arising in the bone is an extremely rare condition [1]. A primary intraosseous squamous cell carcinoma (PIOSCC) is defined as ‘a squamous cell carcinoma arising within the jaw bones, which has no initial connection with the oral mucous membrane and develops from remnants of odontogenic epithelium deriving embryologically from ledge or epithelial cell rest of malassez’ [2]. Most of them arise in odontogenic cysts such as residual periapical cysts, dentigerous cysts, and odontogenic keratocysts (OKCs), such lesions are called PIOSCC *ex *odontogenic cyst [1]. Of these cysts, OKC linings seem to have higher mitotic activity than those of other odontogenic cysts, with a great potential to evolve into squamous cell carcinoma (SCC), and although rare, it has been reported [3].

This article reports a unique case of a 20-year-old female patient who presented with PIOSCC arising from a pre-existing OKC in the posterior mandible.

## Case report

A 20-year-old female patient reported with a complaint of mild, dull pain and swelling of the left lower jaw for the several months. The patient was apparently normal a few months prior to examination, but then she noticed a pea-sized swelling in the lower left posterior jaw. Eventually, over a period, the swelling enlarged to the present size and was associated with a dull, aching pain. She took anti-inflammatory drugs for symptomatic relief, but they were ineffective, with no improvement.

Her medical history was insignificant. Her dental history revealed the extraction of her lower left third molar two years before. The patient’s family and personal history was non-contributory. On physical examination, She was healthy and her blood parameters were within normal limits.

**Figure 1:**
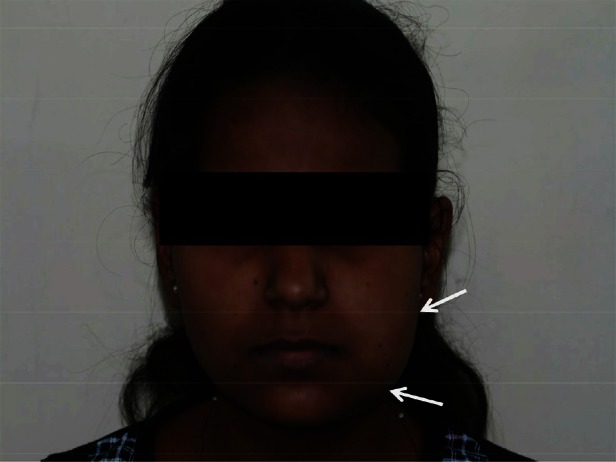
Extraoral photograph showing diffuse swelling on left side of the mandible, with facial asymmetry

Extra-oral examination showed an oval-shaped swelling on the facial aspect of the left mandible, with facial asymmetry (Figure 1). On inspection, the swelling measured around 5 × 3 cm in size, extending from the left corner of the lip to the left pre-auricular region posteriorly and inferiorly to the inferior border of mandible. The skin over the swelling was stretched, and the surrounding tissues appeared to be normal. No scars, sinuses, ulcerations, or discoloration were detected over the swelling. On palpation, there was no local rise in temperature. The swelling was non-tender, firm in consistency, non-fluctuant, non-reducible and non-compressible. The regional lymphadenopathy was also absent.

**Figure 2:**
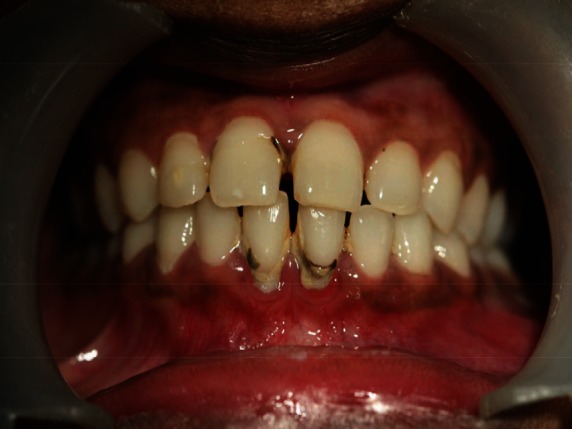
Intraoral photograph showing obliteration of left buccal sulcus

Intraoral soft tissue examination revealed obliteration in the left posterior vestibular region of mandible (Figure 2). The overlying alveolar mucosa was intact without any evidence of a mass or ulcer. There was no nerve parasthesia. Hard tissue examination revealed deep proximal caries with the lower left first molar.

**Figure 3:**
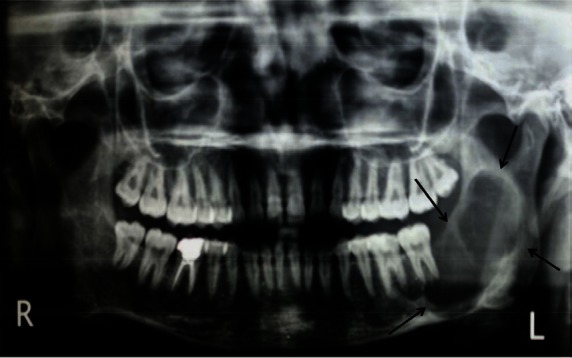
Panoramic radiograph showing multilocular radiolucent lesion in ascending ramus

**Figure 4:**
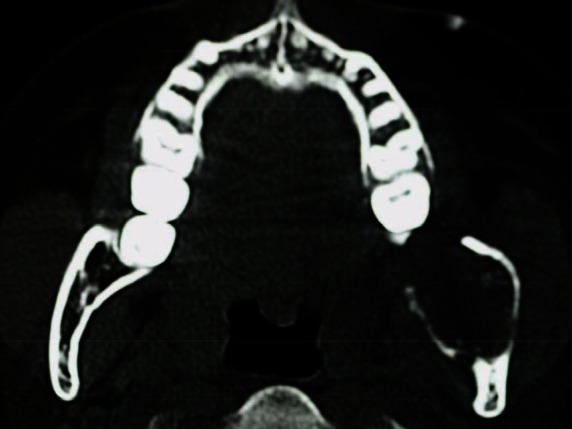
CT scan showing expansion and perforation of buccal and lingual cortical plates

An orthopantomogram demonstrated a multilocular radiolucent lesion with sclerotic margin, which extended from the distal root of the lower left first molar to the sigmoid notch of the ascending ramus of the left mandible (Figure 3). On the postero-anterior (PA) view, the lesion showed marked radiolucency in the left posterior mandible. The computerized tomography (CT) scan also suggested expansion and thinning of both the buccal and lingual cortical plates (Figure 4). Based on the history and clinical as well as radiographic examination, a provisional diagnosis of OKC, ameloblastoma, ameloblastic fibroma, intraosseous mucoepidermoid carcinoma was given.

A bone biopsy with trocar was not performed as there was a risk of bone fracture. An excisional biopsy was performed. Taking into consideration the provisional diagnosis of OKC, Carnoy’s solution was directly applied following excision to prevent recurrence. A careful general physical examination was carried out to rule out distant metastasis.

**Figure 5:**
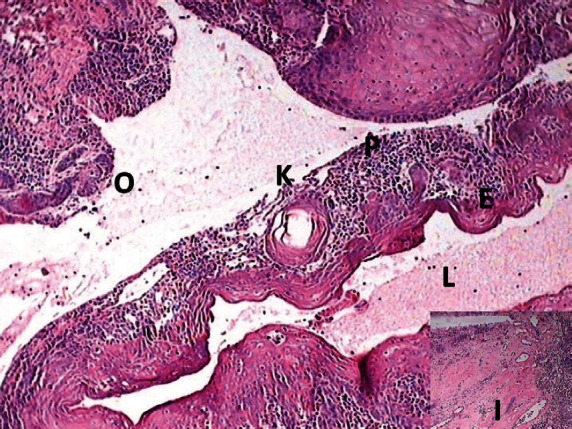
Photomicrograph showing the transition of OKC into SCC (lumen (L), epithelial lining (E), keratin pearl (K), epithelial lining proliferating into connective tissue capsule (P), odontogenic islands (O), and inductive changes (I, inset))

**Figure 6:**
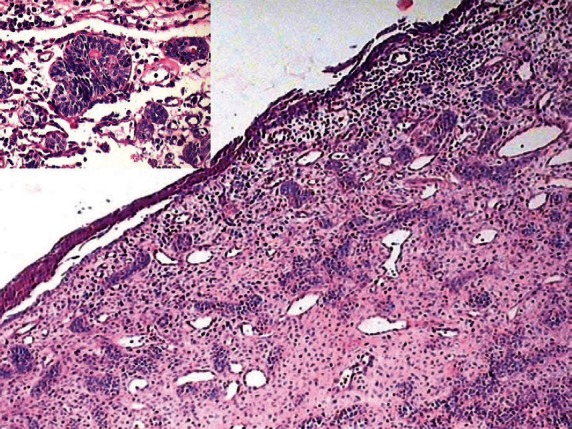
Odontogenic epithelium showing mural proliferation in the form of odontogenic islands. The inset shows the odontogenic islands at a higher magnification

**Figure 7:**
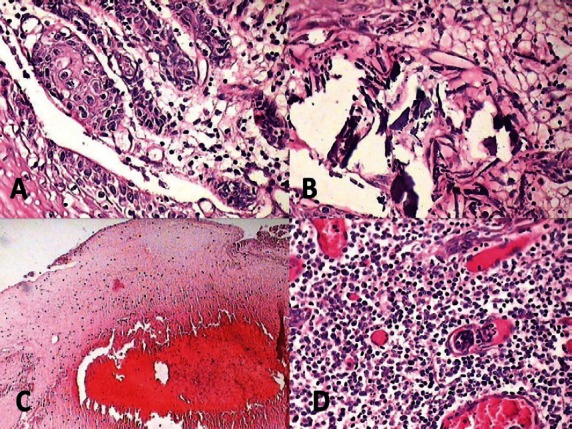
Photomicrograph showing features of malignancy: (A) odontogenic islands with mitotic figures, (B) dystrophic calcification, (C) areas of necrosis, and (D) angiogenesis associated with odontogenic islands

**Figure 8:**
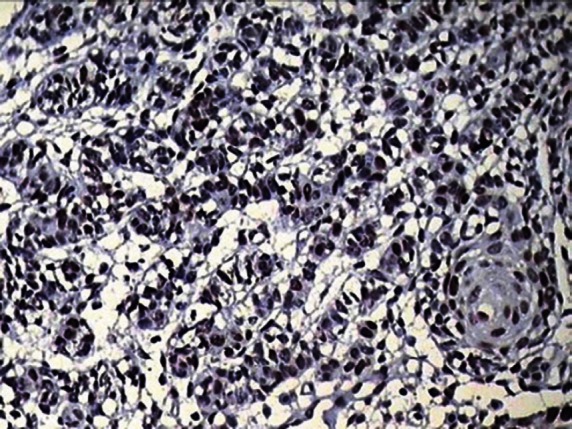
Photomicrograph showing positive p53 staining in the invading odontogenic islands

Histopathological examination with haematoxylin and eosin staining (Figures 5–7) showed a proliferating odontogenic epithelial lining with dysplastic features in the basal cell layer in the form of hyperchromatism, pleomorphism, basal cell hyperplasia, bizarre mitotic figures, and an altered nucleocytoplasmic ratio. The epithelial lining also showed proliferation within the connective tissue capsule in the form of islands and nests with palisading of tall columnar cells. At many places, keratin pearl formation and individual cell keratinisation was also seen within the islands, resembling well-differentiated SCC. The lesional stroma showed inductive changes around the odontogenic islands. The numerous dystrophic calcifications with features of angiogenesis were also suggestive of malignancy. Mucicarmine staining was performed to rule out the diagnosis of intraosseous (central) mucoepidermoid carcinoma. The histopathological findings were suggestive of PIOSCC arising from an OKC. Immunohistochemical staining (Figure 8) helped to confirm the diagnosis, with the aggressive epithelial component stained intensely and diffusely positive with p53.

Compiling the information obtained by the radiological, histopathological, histochemical, and immunohistochemical investigation, a final diagnosis of PIOSCC was made. The patient was referred to the regional oncology centre for further evaluation and management. The case was categorised as pT3pN0 and was considered for surgical treatment and concomitant radiotherapy. A hemimandibulectomy was supposed to be performed from the left premolar region to the subcondilar region along with functional head and neck dissection of the respective lymph nodes. Tracheostomy and reconstruction with osteomyocutaneous free fibular flap was also planned, but unfortunately, the patient did not return for further evaluation or treatment.

## Discussion

PIOSCC was first described by Loos in 1913 as a central epidermoid carcinoma of the jaw [4]. Wills in 1948 renamed it an intraalveolar epidermoid carcinoma [5]. It was then termed primary intraalveolar epidermoid carcinoma by Shear 1969 [6]. The World Health organization (WHO) and Pindborg approved the term ‘primary intraosseous odontogenic carcinoma (PIOC)’ in 1972 and classified the lesion as an odontogenic carcinoma [7]. Subsequently, Elzay (1982) modified the WHO classification on PIOC of the jaw [8]. Slootweg and Muller slightly modified Elazy’s classification in 1984 by considering various possible aetiological factors [9]. Waldron and Mustoe (1989) completed the classification by addition of intraosseous mucoepidermoid carcinoma (IMEC) as a fourth type of PIOC [10]. Finally, in the new WHO classification published in 2005, PIOSSC replaced the old terms and is sub-classified as (i) a solid tumour that invades marrow spaces and induces bone resorption, (ii) SCC arising from an OKC lining or carcinoma arising in other odontogenic cysts, and (iii) SCC associated with benign epithelial odontogenic tumours [11].

According to the new WHO classification of odontogenic cysts and tumours (2005), OKCs were designated as keratocystic odontogenic tumours (KCOTs). PIOSSC arising from the wall of an OKC or KCOT is a rare tumour occurring within the jaw bones [12]. Bodner *et al *conducted a retrospective study of 116 cases of PIOSCC between 1938 and 2010. The results of the study showed that there have been only 16 known cases of PIOSCC arising from an OKC [2].

The pathogenesis of PIOSCC arising from an OKC is largely unknown. According to Browne and Gouch [13], keratin metaplasia followed by epithelial hyperplasia and then epithelial dysplasia of cyst epithelia were the significant events in the development of SCC in OKCs. Therefore, van der wal *et al *mentioned that the presence of keratinization in the cyst lining results in a greater risk for malignant changes [14]. According to Gardner [15] and Yu *et al *[16], long-standing chronic inflammation has also been suggested as a possible predisposing factor. The main mechanism of the inflammation-induced carcinogenesis includes the formation of reactive oxygen metabolites, causing damage to DNA, protein, and cell membranes and eventually showing compensatory proliferative response of neoplastic cells against the normal apoptotic mechanism [2]. As in the present case the patient had no genetic predisposition nor did she have any abusive habits, the long-standing inflammation following the extraction of a third molar two years before presentation might be the main causative factor for the malignant transformation. Some authors speculated that the diagnostic delay could range from 2 to 36 weeks [17]. Similarly, as PIOSCC is an asymptomatic lesion at its early stages, there could be a delay in diagnosis.

Gardner (1975) proposed definitive criteria to identify a lesion as PIOSCC *ex *odontogenic cyst: (i) a microscopic transition area from benign cystic epithelial lining to SCC, (ii) an intact overlying oral mucosa, (iii) absence of carcinoma in the adjacent structures, and (iv) absence of metastatic carcinoma from a distinct tumour [10]. Our case fulfills all of the above-mentioned criteria.

A review of the literature regarding PIOSCC showed the mandible (79%) as the predominant site of occurrence as compared with the maxilla (21%) [2, 11–13]. Similar data were recorded in the present case. According to the literature, PIOSCC arising from an OKC shows a wide age range, with a mean age of 57 years, and the male–female ratio is 2:1. Similar findings were observed in studies conducted by Aboul-hosn Centenero *et al *[18], Mosqueda-Taylor *et al *[19] and Scheer *et al *[20]. But our case was reported in a 20-year-old woman, which made it unique.

Clinically, the patient showed the symptoms of the early stage of PIOSCC as pain, swelling, and destruction of buccal and lingual cortical plates with an intact overlying oral mucosa, which were in accordance with studies reported by others [10, 21–23], with an absence of lymphadenopathy, sensory disturbances such as parasthesia and numbness due to nerve compression.

Radiographic examination is one of the most effective methods to detect PIOSCC [11]. Benign odontogenic cysts can be considered during a differential diagnosis for PIOC. In our case, the patient showed well-defined multilocular radiolucent areas, more or less rounded with a scalloped margin or multiloculated with sclerotic border, almost extending from the distal root of the lower first molar to the sigmoid notch on the left side of the ramus of the mandible. Therefore, well-circumscribed unilocular cystic lesions such as radicular cysts, dentigerous cysts and calcifying odontogenic cysts were excluded. According to Kaffe *et al *(1998), PIOSCC shows great variation, presenting as a multilocular lesion with an ill- or well-defined but non-corticated borders radiographically. Our data are in accordance with this statement. The PA view shows a marked destruction of the body as well as the ramus of the left mandible.

In OKCs, there is almost non or very little expansion seen in the cortical plates on axial CT scan view [12]. But in our case, on CT, intraosseous carcinomas usually showed an extensive buccolingual expansion of cortical plates [25, 26]. The probability of a malignant lesion is due to the presence of lingual expansion [26] as well as jagged or irregular margins with indistinct borders, which was seen in our case. Therefore, when such a lesion is asymptomatic and casually discovered, one should correlate with the radiographic features of malignancy.

The majority of cases of PIOSCC arising from an OKC represent well-differentiated SCCs. Evidence of a cystic component is a prerequisite for the diagnosis.

The histopathologic criteria employed to document an odontogenic origin of lesion are (i) malignant transformation of the cyst lining, with a transition from the normal lining epithelium to dysplasia and to carcinoma, (ii) palisaded columnar cells, and (iii) inductive influence of connective tissue. All of these features are seen in our case. Histopathologic characteristics reminiscent of ameloblastoma, such as alveolar or plexiform patterns and peripheral palisading of cells, may be exhibited in PIOSCC solid type; nonetheless, typical features of ameloblastic differentiation, which would justify a diagnosis of ameloblastic carcinoma, are lacking [8].

Benign and malignant tumours of odontogenic epithelium as well as SCC of mucosal origin and metastatic tumours to jaw bones should be considered in the differential diagnosis.

Most benign odontogenic epithelial tumours such as acanthomatous ameloblastomas and squamous odontogenic tumours, in addition to features specific to each one of them, share a lack of invasive growth, with the exception of ameloblastoma and, to a lesser extent calcifying epithelial odontogenic tumor (CEOT), which may be locally invasive, but benign CEOT histologically demonstrates sheets of polygonal cells with ample eosinophilic cytoplasm, distinct cell borders and very conspicuous intercellular bridges. In addition, other diagnostic features are amyloid deposition and Liesegang ring calcifications. Therefore, these findings allow discrimination between PIOSCC and CEOT [27].

Malignant tumours of the odontogenic epithelium, including ameloblastic carcinomas, intraosseous mucoepidermoid carcinomas, clear cell odontogenic carcinomas, and odontogenic ghost cell carcinomas, should also be ruled out.

Ameloblastic carcinoma demonstrates malignant features of conventional ameloblastoma, that is, bland cytological features with nuclear palisading, reverse nuclear polarization o the nuclei, and vacuolization of the cytoplasm within the tumour islands; these were absent in our case of PIOSCC [28].

The absence of a mucous cell component in PIOSCC, verified by a negative mucicarmine staining that we performed, serves to distinguish it from intraosseous mucoepidermoid carcinoma [29].

The nests and strands, intermingled with smaller islands of clear cells and eosinophilic polygonal cells, are always considered the histological hallmark of clear cell odontogenic carcinoma [30]. In contrast, PIOSCC exhibits sheets or islands of malignant epithelial cells, with an absence of a clear cell component.

Odontogenic ghost cell carcinoma is a tumour showing the microscopic features of a benign calcifying odontogenic cyst or tumours, including the malignant epithelial component along with the presence of ghost cells and calcified material, which are completely absent in PIOSCC [31].

The SCC of mucosal origin is also ruled out, as this lesion is unlikely to develop in the younger age group, as in our case. Also, malignant histopathologic changes observed in this cyst confirmed the diagnosis of PIOSCC [12].

The exclusion of metastasis is based on the absence of a initial connection with an ulcer in the overlying mucosa as well as a distinct primary tumour in a six-month follow-up period [32]. Both the findings were absent in our case.

 Finally, the aggressiveness of PIOSCC was confirmed with immunohistochemical proliferative marker p53 with a strong positivity.

The treatment for PIOSCC is principally wide local resection, and in most cases, enbloc excision or radical resection of the involved bone is also advised [33]. McGregor and MacDonald (1993) suggested complete excision of the mandibular canal and its contents with no conservative approach to resection of the ramus in the case of tumour infiltration in the posterior mandible. In our case, the osteomyocutaneous free microvascular graft of fibula is the treatment of choice to reconstruct three-dimensionally the hemimandible, maintaining the functionality and the aesthetical profile. Keeping the mandibular condyle allows the patient to preserve the temporomandibular joint, which gives a better mobility to the rest of the jaw and also diminishes the risk of post-operative pain [18]. In general, 50% of cases of PIOSCC show metastasis to the cervical lymph nodes; therefore, radiation and chemotherapy may also be performed as adjunctive treatments along with the surgical excision [29]. According to the literature, its prognosis is relatively poor, with a five-year survival rate ranging from 30% to 40% [25].

## Conclusion

This unique case report of PIOSCC presents a comprehensive review of the literature, origin, clinical manifestations, radiographic, and histopathologic features with malignant transformation of an OKC in the mandible of a young female patient. It also highlights the importance of careful histopathological examination of apparently innocuous odontogenic cysts because of the possibility of carcinomatous change in their epithelial lining.
